# Supplementary material for: PTCH1 Gene Variants, mRNA Expression, and Bioinformatics Insights in Mexican Cutaneous Squamous Cell Carcinoma Patients
Source: Biology (Basel). 2024 Mar 16;13(3):191. doi: 10.3390/biology13030191 (PMC10968426; doi:10.3390/biology13030191)
Supplement: Supplementary file 1 [file biology-13-00191-s001.zip › biology-2893448-supplementary.pdf]

```
1            11           21           31           41
MASAGNAAEP   QDRGGGGSGC   IGAPGRPAGG   GRRRRTGGLR   RAAAPDRDYL
eeeeeebeee   eeeeeebeee   eeeeeeeeee   eeeeeeeeee   eeeeeeeeeb

51           61           71           81           91
HRPSYCDAAF   ALEQISKGKA   TGRKAPLWLR   AKFQRLLFKL   GCYIQKNCGK
eeeeebebbb   bbeebeeeee   eeeebebbbe   bebeebbbeb   bbebeeeebbe
                    f f        f   f   s  f   s s   ss sf fssf

101          111          121          131          141
FLVVGLLIFG   AFAVGLKAAN   LETNVEELWV   EVGGRVSREL   NYTRQKIGEE
bbbbbbbbbb   bbbbbbbbbb   beeebeebbb   ebeeebeeeb   eebeeeeeee
 s  ss       s ssss  s     ff  f s s   f  ffsf f     fs  f fff

151          161          171          181          191
AMFNPQLMIQ   TPKEEGANVL   TTEALLQHLD   SALQASRVHV   YMYNRQWKLE
bbbbbebbbe   eeeeeeeebb   bbebbeeebe   ebbeebebeb   bbbeeebebe
     f ssf   f      f s   s      f     fs  fs             s s

201          211          221          231          241
HLCYKSGELI   TETGYMDQII   EYLYPCLIIT   PLDCFWEGAK   LQSGTAYLLG
ebbeeeeeeb   beebbbeebb   eebbebbbbb   bbebbbeebe   eeebebebee
  s ffff      f               fs   s   ssf    f f   f  s

251          261          271          281          291
KPPLRWTNFD   PLEFLEELKK   INYQVDSWEE   MLNKAEVGHG   YMDRPCLNPA
eeebebbebe   eeebbeebee   beeebeebee   bbeebeebbb   bbeeebeeee
       f f   f                            fs f  s   ss f sf f

301          311          321          331          341
DPDCPATAPN   KNSTKPLDMA   LVLNGGCHGL   SRKYMHWQEE   LIVGGTVKNS
eeebeeeeee   eeeeeeeebb   eebebebeee   beebbebeee   bbbebeeeee
f  sf    f   f              s sfs ff   s f s sfff   ss f

351          361          371          381          391
TGKLVSAHAL   QTMFQLMTPK   QMYEHFKGYE   YVSHINWNED   KAAAILEAWQ
eeebbbbebb   ebbbebbbee   ebeeebeeee   ebbebebeee   ebbbbbebbe
      s ss   f   fsssff   fs ff             fsf     fs  ssf sf

401          411          421          431          441
RTYVEVVHQS   VAQNSTQKVL   SFTTTTLDDI   LKSFSDVSVI   RVASGYLLML
eebbeebeeb   beeeeeeebb   bbebbebeeb   beebbebbbb   ebbbbbbbbb
f               f  f       sf sf  fs     fss  s     f   sss ss

451          461          471          481          491
AYACLTMLRW   DCSKSQGAVG   LAGVLLVALS   VAAGLGLCSL   IGISFNAATT
bbbbbbbbeb   ebeeeeebbb   bbbbbbbbbb   bbbebbbbbb   bebbbebbbe
ssssss sf    fs  fffsss   s ss ss ss   ss fssssss    f ssfsssf

501          511          521          531          541
QVLPFLALGV   GVDDVFLLAH   AFSETGQNKR   IPFEDRTGEC   LKRTGASVAL
ebbbbbbbbb   bbbebbbbbb   bbeeeeeeee   bebeeeeeeb   beeeebebbb
fsss sssss   sssf     s    sff          f   ffffs   s fffsfs s

551          561          571          581          591
TSISNVTAFF   MAALIPIPAL   RAFSLQAAVV   VVFNFAMVLL   IFPAILSMDL
bbbeebbbbb   bbbbbbbebb   bbbebebbbb   bbbbbbbbbb   bbbbbbbbbb
ss ff  ss    sss  ssf     s sf f              s       sss s ss
```

601	611	621	631	641
YRREDRRLDI	FCCFTSPCVS	RVIQVEPQAY	TDTHDNTRYS	PPPPYSSHSF
eeeeeeebeb	bbbbbbebbe	ebbebeeeeb	eeeeeeeeee	eeeeeeeeeb
ff f f	ss			
651	661	671	681	691
AHETQITMQS	TVQLRTEYDP	HTHVYYTTAE	PRSEISVQPV	TVTQDTLSCQ
eeebebebee	ebebeeebee	eeeebbbbbe	eeeebeeeee	eeeeeeeeee
s f f	f f	s		
701	711	721	731	741
SPESTSSTRD	LLSQFSDSSL	HCLEPPCTKW	TLSSFAEKHY	APFLLKPKAK
eeeeeeebee	bbeebeeeee	ebeeeebbeb	ebbebbeeeb	bebbeeeeee
f ff	s f	s	f sf s	sf sf f
751	761	771	781	791
VVVIFLFLGL	LGVSLYGTTR	VRDGLDLTDI	VPRETREYDF	IAAQFKYFSF
bbbbbbbbbb	bbbbbebbbe	beebbebbbb	beeeeeebeb	bebebebbbb
s s s	s s fsss	s fss ssss	sff f f s	sf ss
801	811	821	831	841
YNMYIVTQKA	DYPNIQHLLY	DLHRSFSNVK	YVMLEENKQL	PKMWLHYFRD
bbbbbbbeee	eeeebeebbe	ebeeebeebe	bbbeeeeeeb	eebbbbbbee
s s s	f f s	f s		f s s
851	861	871	881	891
WLQGLQDAFD	SDWETGKIMP	NNYKNGSDDG	VLAYKLLVQT	GSRDKPIDIS
bbeebeebbe	eebeebebee	eeeeeeeeeb	bbbbebbbee	eeeeeeeebe
ss f sf	f s	fff f	sssfss ff	f f
901	911	921	931	941
QLTKQRLVDA	DGIINPSAFY	IYLTAWVSND	PVAYAASQAN	IRPHRPEWVH
ebbeeebbee	eebbbeebbb	bbbbbbbeee	ebbbbbeeee	beeeeeeebe
	f	ss fff	fs s ffff	f f f
951	961	971	981	991
DKADYMPETR	LRIPAAEPIE	YAQFPFYLNG	LRDTSDFVEA	IEKVRTICSN
eeeeeeeebb	bebeeeeebe	ebebbbbbeb	beebbebbeb	beebebbbee
f ff	sf fff f	f fsssss	s s fs f	sffsf s f
1001	1011	1021	1031	1041
YTSLGLSSYP	NGYPFLFWEQ	YIGLRHWLLL	FISVVLACTF	LVCAVFLLNP
bbeeebeeee	eeebbbbbee	bbebbbbbbb	bbbbbbbbbb	bbbbbbbbbb
f ff	fffss ssff	s sss s	s sss	ss ss
1051	1061	1071	1081	1091
WTAGIIVMVL	ALMTVELFGM	MGLIGIKLSA	VPVVILIASV	GIGVEFTVHV
bebbbbbbbb	bbbbbbbbbb	bbbbbbebeb	bbbbbbbbbb	bbbbebbbbb
sfs ss s	s s ssss	sss sfsfs	ssssss sss	ssssfssss
1101	1111	1121	1131	1141
ALAFLTAIGD	KNRRAVLALE	HMFAPVLDGA	VSTLLGVLML	AGSEFDFIVR
bbbbbbbbee	eeeebbbbbe	ebbbbbbbeb	bbbbbbbbbb	bbbebebbbe
ss sss f	f ssf	f ssss sfs	ss ssssss	sssfsfss
1151	1161	1171	1181	1191
YFFAVLAILT	ILGVLNGLVL	LPVLLSFFGP	YPEVSPANGL	NRLPTPSPEP
bbbbbbbbbb	bbbbbebbbb	bbbbbebbee	eeeeeeeeee	eeeeeeeeee
s ss ss	fss	s sf f		

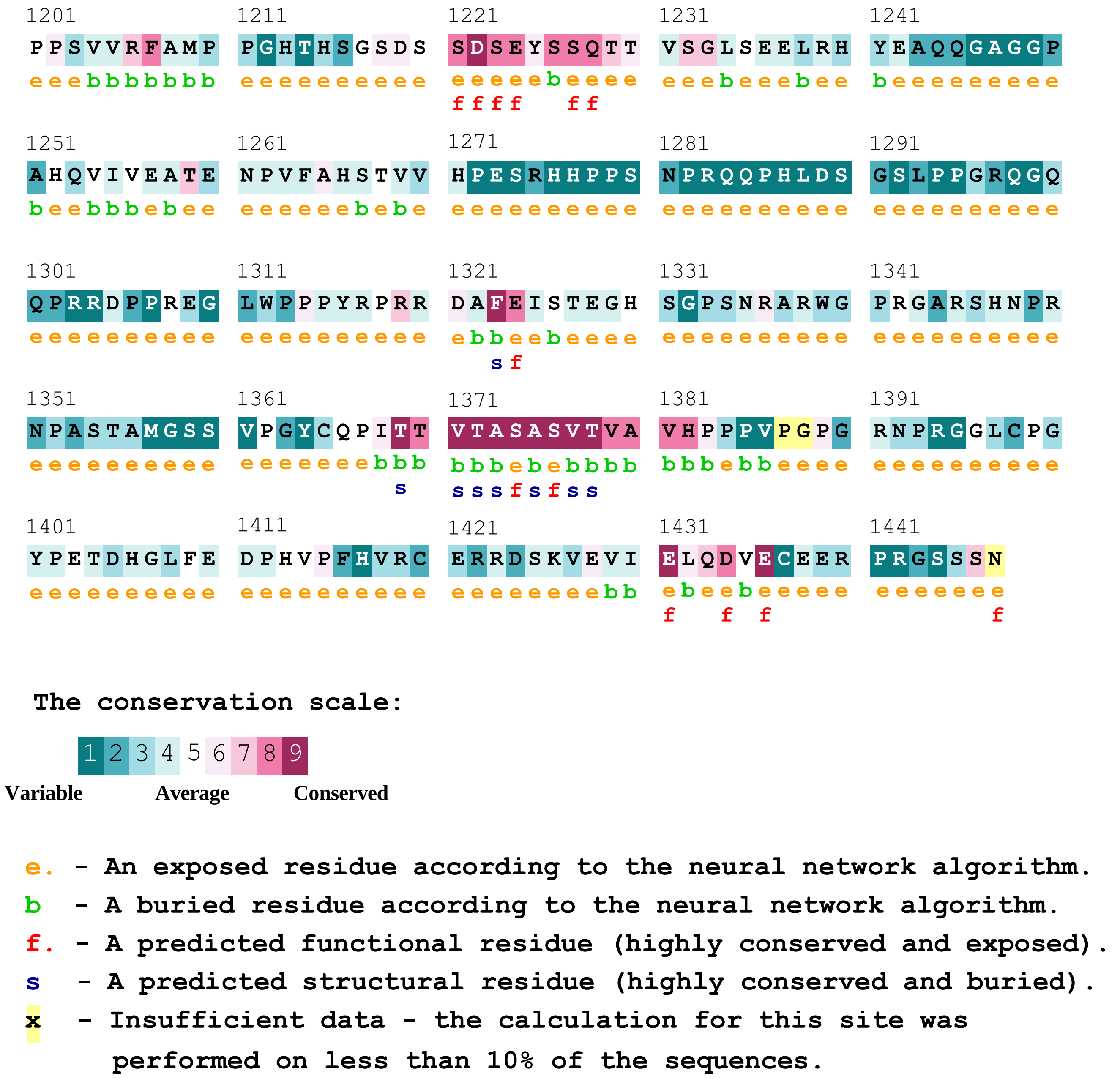

Figure S1. The complete sequence of PTCH1 analyzed using the Consurf platform.
